# Chromatin Switches during Neural Cell Differentiation and Their Dysregulation by Prenatal Alcohol Exposure

**DOI:** 10.3390/genes8050137

**Published:** 2017-05-11

**Authors:** David P. Gavin, Dennis R. Grayson, Sajoy P. Varghese, Marina Guizzetti

**Affiliations:** 1 Jesse Brown Veterans Affairs Medical Center, 820 South Damen Avenue (M/C 151), Chicago, IL 60612, USA; sajoy.varghese@va.gov; 2 Center for Alcohol Research in Epigenetics, Department of Psychiatry, University of Illinois at Chicago, 1601 W. Taylor St., Chicago, IL 60612, USA; dgrayson@psych.uic.edu; 3 Department of Behavioral Neuroscience, Oregon Health & Science University, 3181 SW Sam Jackson Park Road L470, Portland, OR 97239, USA; 4 Veterans Affairs Portland Health Care System, 3710 Southwest US Veterans Hospital Road, Portland, OR 97239, USA

**Keywords:** histone modifications, DNA methylation, fetal alcohol, neural cell differentiation, neurons, astrocytes, chromatin

## Abstract

Prenatal alcohol exposure causes persistent neuropsychiatric deficits included under the term fetal alcohol spectrum disorders (FASD). Cellular identity emerges from a cascade of intrinsic and extrinsic (involving cell-cell interactions and signaling) processes that are partially initiated and maintained through changes in chromatin structure. Prenatal alcohol exposure influences neuronal and astrocyte development, permanently altering brain connectivity. Prenatal alcohol exposure also alters chromatin structure through histone and DNA modifications. However, the data linking alcohol-induced differentiation changes with developmental alterations in chromatin structure remain to be elucidated. In the first part of this review, we discuss the sequence of chromatin structural changes involved in neural cell differentiation during normal development. We then discuss the effects of prenatal alcohol on developmental histone modifications and DNA methylation in the context of neurogenesis and astrogliogenesis. We attempt to synthesize the developmental literature with the FASD literature, proposing that alcohol-induced changes to chromatin structure account for altered neurogenesis and astrogliogenesis as well as altered neuron and astrocyte differentiation. Together these changes may contribute to the cognitive and behavioral abnormalities in FASD. Future studies using standardized alcohol exposure paradigms at specific developmental stages will advance the understanding of how chromatin structural changes impact neural cell fate and maturation in FASD.

## 1. Introduction

A missing link in the study of fetal alcohol spectrum disorders (FASD) is whether the effects of prenatal alcohol exposure on neural cell fate are attributable to the effects of alcohol on chromatin [1]. Neural cell differentiation (neuroepithelial cells to radial glia, and radial glia to neurons and macroglia (astrocytes and oligodendrocytes)) is dependent upon extracellular cues received by developing cells, and whether the cells receiving these signals are able to respond by changing their transcriptional profiles [2,3,4]. Transcriptional control of the production of many of these extracellular signaling proteins, as well as the transcriptional responses of the targeted cells, is partly dependent upon chromatin architecture [5,6,7]. Recent studies indicate that prenatal alcohol exposure affects neural cell differentiation, maturation, and chromatin architecture. However, how the chromatin structural changes observed in FASD models mediate altered neural cell development have yet to be fully elucidated.

The overall scope of this review article is to discuss epigenetic changes that occur during normal neurodevelopment, and the impact of prenatal alcohol exposure on chromatin structure in relation to neural cell fate and maturation. We particularly emphasize future research directions that will lead to a better understanding of the relationship between alcohol-induced chromatin modifications, neural cell fate determination, cell maturation, and FASD.

## 2. Chromatin Structure and Gene Expression

The definition of “epigenetics” has gone through several etymological stages. In the 1940s, Waddington originally defined epigenetics as “the branch of biology which studies the causal interactions between genes and their products which bring the phenotype into being” [8]. In other words, Waddington’s definition of epigenetics referred to the combined effects of genotype and gene expression on phenotype. In 1958, David Nanney used the term to mean inherited phenomena not explained by conventional genetics [9]. To this day, a consensus regarding the definition of epigenetics remains elusive. The broadest definition includes any factors that affect gene transcription, such as noncoding RNAs, repressor proteins, histone modifications, etc. [10,11], while the narrower definition includes only DNA modifications and perhaps a few histone protein post-translational modifications (PTMs) that propagate following cell division [12,13,14]. We do not take a position on whether a broad or narrow definition of epigenetics is more appropriate. In the current review, we use the term to refer to histone protein PTMs and DNA modifications.

Any mediator of long-lasting changes in cell fate must have a high degree of stability. DNA methylation is mitotically and meiotically heritable and has no requirement for turnover in non-dividing cells, such as most neurons and astrocytes [15,16]. Histone proteins have a half-life of months, and there is evidence that, for example, H3K4 and H3K27 methylation are mitotically inherited [13,17]. Histone and DNA methylation are therefore plausible candidates for participating in the lifelong manifestations of FASD. Some histone acetylation marks have a half-life that is only minutes long [12]. This makes acetylation a poor candidate to be responsible for enduring illnesses such as FASD. Acetylation is included in this discussion because it is a marker of chromatin structure, and is tightly correlated with more stable and longer-lasting marks, such as H3K4 methylation and DNA methylation [18]. While non-coding RNAs, including microRNAs, modulate signaling pathways involved in cell fate determination and brain development and are altered by ethanol, they are relatively short-lived. The effects of alcohol on non-coding RNA are reviewed elsewhere [19]. This review focuses on the more stable epigenetic mediators that directly affect chromatin conformation through histone modifications and DNA methylation.

Chromatin structure does not dictate transcriptional activity; rather, the evidence suggests that it increases or decreases the accessibility of transcription factors to DNA [20]. Restrictive chromatin prevents transcription by barring transcription factors and RNA polymerases access to DNA. Conversely, less restrictive chromatin allows gene expression to occur. Histone PTMS can be broadly categorized as being associated with less or more restrictive chromatin. Histone H3 and H4 lysine acetylation and H3 lysine 4 methylation (H3K4me2/3) are usually associated with a transcriptionally permissive chromatin structure [21], while dimethylated H3 lysine 9 (H3K9me2) and di- and trimethylated H3 lysine 27 (H3K27me2/3) are associated with lower levels of gene expression. 

The enzymes and attendant proteins that regulate histone acetylation and H3K4 methylation are important factors for maintaining these PTMs and their effects on transcription. Histone H3 and H4 acetylation is catalyzed by histone acetyltransferases (HATs), including cAMP response element-binding protein binding protein (CREBBP) and E1A binding protein p300 (EP300). Histone deacetylases (HDACs) remove this PTM [22]. H3K4 methylation is catalyzed by lysine methyltransferase-2A (KMT2A), ASH1L, and SET Domain Containing Lysine Methyltransferase (SETD7), among others. This mark is erased by the lysine demethylases Lysine Demethylase 1A (KDM1A), KDM1B, and KDM5D. 

The enzymes that add and subtract histone marks associated with transcriptional repression, such as H3K9me2 and H3K27me3, are also important in regulating gene expression. Euchromatic Histone-Lysine N-Methyltransferase-1 (EHMT1), EHMT2, and SET Domain Bifurcated 1 (SETDB1) enzymes catalyze H3K9 methylation [23]. H3K27 methylation is catalyzed by the Polycomb Repressive Complex 2 (PRC2), which is comprised of Embryonic Ectoderm Development (EED), Enhancer of Zeste 1 (EZH1), and EZH2, among others [24]. Following H3K27 methylation, PRC1, which contains Ring finger protein 1 (RING1) and Ring finger protein 2 (RNF2), among others, is recruited where it participates in forming heterochromatin [25]. Together PRC1 and 2 are termed Polycomb Group (PcG) proteins. 

Methylated cytosine nucleotides (5-methylcytosine (5mC)) are also often components of heterochromatin. 5mC can be further modified through the successive oxidation of the methyl group forming hydroxymethyl, formyl, or carboxyl groups [26]. This review focuses on the best-characterized and most abundant DNA modifications in the brain, 5mC and 5-hydroxymethylcytosine (5hmC). 5mC is catalyzed by members of the DNA methyltransferase family of enzymes; DNMT1, 3A, and 3B [27]. Generally, 5mC is associated with lower transcriptional activity. On the other hand, there are reports of a positive correlation between gene expression and gene-body 5mC levels [28], including at neuronal genes [29]. 5mC serves as a ligand for methyl-CpG-binding domain proteins (MBD), including MeCP2 [30]. 5mC is stable, but can be converted to 5hmC by Ten-eleven Translocation (TET) enzymes [31]. Several studies indicate that 5hmC is a necessary intermediary step between 5mC and nonmethylated cytosines as part of a base excision repair DNA demethylation process [32]. 5hmC within gene bodies in the brain is generally associated with active transcription, while when it is located within promoter regions it is associated with decreased gene expression [33,34,35,36,37,38,39]. More recently, 5hmC has been reported as the predominant modification located within enhancers poised for activation [28]. Overall, however, the association between 5hmC levels and gene expression in the brain is not entirely clear. This suggests that 5hmC may serve additional functions, such as influencing RNA splice choices [40]. 

Traditionally it was believed that cytosine bases could only be methylated when they preceded guanine bases (CpG). However, recently it was demonstrated that methylated CpHs (where H is not guanine) may be equal in number to methylated CpGs, specifically in the brain [39]. Methylation of CpH is catalyzed by DNMT3A and serves as a ligand for MeCP2 binding [41]. 

## 3. The Pluripotency/Neurogenesis Switch 

Chromatin structure plays an important role in maintaining pluripotency prior to cellular differentiation and attainment of a neural cell fate. This occurs through maintaining the continued expression of stemness genes such as POU domain, class 5, transcription factor 1 (*Pou5f1*), *Nanog*, and SRY (sex determining region Y)-box 2 (*Sox*2) [42,43,44]. These stemness genes participate in preventing the premature expression of neural genes. Independent of these factors, H3K27 methylation also prevents the premature differentiation of pluripotent cells.

Pluripotency is associated with higher H3K4 methylation and histone acetylation levels globally, and at specific stemness gene promoters [45,46,47]. The transcription factor *Pou5f1* appears to be particularly sensitive to fluctuations in histone modifications. Increased *Pou5f1* expression in induced pluripotent cells is associated with high H3K4me2 and histone acetylation and low H3K9me2 at its promoter. Interestingly, in these cells neuronal development genes, Neurofilament, Medium Polypeptide (*Nefm*) and Thymus cell antigen 1, theta (*Thy1*) also acquire higher amounts of H3K4me2 and histone acetylation, but with no concomitant increase in their expression [48]. A possible explanation is that the transcriptional activity of certain genes, such as *Pou5f1*, may be more sensitive to changes in chromatin structure than other genes. In support of this hypothesis, several studies demonstrate a particularly close association between low DNA methylation, high histone acetylation, high histone arginine methylation, and high *Pou5f1* expression [49,50,51].

POU5F1 and NANOG are in part responsible for suppressing the premature expression of neuronal genes in pluripotent cells by increasing RE1-silencing transcription factor (REST) expression [52,53]. REST maintains neuronal genes in a quiescent state through its ability to coordinate a process leading to condensed chromatin at specific promoters. It accomplishes this in association with co-factors, such as REST Co-repressor (RCOR) and SIN3A, as well as the chromatin modifying enzymes, HDAC1 and 2, KDM1A and EHMT1/2, and EHMT2-associated adaptor proteins [54,55,56,57,58,59]. 

A different set of neuronal genes from those silenced by REST are repressed by H3K27 methylation [59,60,61,62]. In fact, the factor(s) responsible for recruiting PcG to specific neuronal gene regulatory regions have not yet been discovered. Thus, the combined effects of REST and PcG protein-coordinated repressor complexes at neuronal gene promoters maintain the pluripotency/neurogenesis switch to a position that favors pluripotency and prevents neural cell differentiation.

After cells attain a neural cell fate, high global H3K27me3 and low global H3K4 methylation repress neuronal differentiation [63,64]. Deletion of the PRC2 component *Ezh2* in the cortex at the onset of neurogenesis, embryonic day (E9.5) in the mouse, results in increased production of cortical neurons by E14 but decreased numbers of neurons at birth [65]. Also, increased expression of the H3K27 demethylase, lysine (K)-specific demethylase 6B (KDM6B), allows for the expression of neurogenesis promoting genes, such as the γ-aminobutyric acid (GABA) ergic neuronal development promoting gene Distal-less homeobox 2 (*Dlx2*) [63,64]. H3K4 methyltransferase KMT2A expression is also necessary for *Dlx2* expression and neurogenesis during development [66]. Thus, any insult that would induce high H3K27 methylation and reduce H3K4 methylation during this period of brain development might suppress neurogenesis and prolong pluripotency.

Based on these reports, pluripotency is maintained prior to neural cell differentiation by the expression of certain stemness proteins that are regulated by changes in chromatin structure. On the other hand, stemness proteins, such as POU5F1 and NANOG, in coordination with REST, recruit the gene silencing chromatin machinery to neuronal genes, preventing premature differentiation. Independent of REST, H3K27 methylation also prevents premature neuronal gene expression by targeting a separate set of neuronal gene promoters. 

## 4. The Neurogenesis/Gliogenesis Switch

During brain development, astrocytes interact with neurons via membrane-bound and soluble factors, providing instructions to neurons leading to neurite outgrowth/dendritic arborization and synaptogenesis [67,68]. In the adult brain, astrocytes regulate many functions; for example, astrocytes reuptake neurotransmitters and K+ at the end of action potentials, provide neurons with substrates for the biosynthesis of neurotransmitters and glucose, control cerebral blood flow, and are involved in the modulation of synaptic transmission by releasing gliotransmitters [69,70].

Pro-astrogliogenesis gene promoters are silenced during the neurogenic phase in part by higher levels of DNA methylation, MeCP2 promoter binding, and promoter H3K9me2, as well as lower levels of transcriptionally facilitative histone modifications, such as H3K4me2/3, acetylated lysine 9 of histone H3 (H3K9ac), and acetylated lysine 14 of histone H3 (H3K14ac) [5,71,72]. 

Histone acetylation plays an important role in the transition from the neurogenic to gliogenic phase of neural cell development. The pro-neuronal transcription factor, Neurogenin 1 (NEUROG1), prevents Phosphorylated Signal Transducer and Activator of Transcription (pSTAT) from activating glial gene transcription by sequestering EP300/CREBBP, a transcription factor with HAT activity, from glia specific promoters [73]. Similarly, global increases in HDAC inhibitor mediated-histone acetylation increase astrogliogenesis and suppress neurogenesis in the E13.5–15.5 ganglionic eminence in astrocyte cultures from postnatal day 2 (P2) rats, and in rats treated from P2 to P4 in the cortex [74,75]. On the other hand, HDAC inhibition in neural stem cells in the E13.5–15.5 cortex and in the adult hippocampus increases the total number of neurons while actively suppressing astrocyte differentiation [75,76,77,78]. One of the genes induced by HDAC inhibitors is the neurotrophic growth factor Brain-Derived Neurotrophic Factor (*Bdnf*), itself an inhibitor of gliogenesis [79,80]. HDAC inhibition also mediates decreases in Glial fibrillary acidic protein (GFAP) expression in primary human astrocytes and in astrocytoma cells [81]. Taken together, it appears possible that the effects of histone acetylation on neuronal vs. astrocyte differentiation depend upon the developmental stage. Histone acetylation appears to amplify ongoing cellular processes, such that increasing histone acetylation promotes neuronal production and inhibits gliogenesis during a period of neurogenesis, while during astrogliogenesis it amplifies the expansion of this cell type.

Histone methylation is also important for the neuronal-glial switch. H3K9me3 and trimethylated lysine 36 of histone 3 (H3K36me3) demethylase Lysine Demethylase 4A (KDM4A) forms a complex with the transcriptional repressor of glial cell differentiation, Nuclear receptor co-repressor (NCOR) [82]. Knockdown of KDM4A leads to a decrease in neuronal differentiation and an increase in astrocytes due to a lack of H3K9me3 demethylation at the *Bdnf* promoter and an increase in H3K36me3 at the *Gfap* promoter [83]. Maintaining low levels of PcG proteins, such as RNF2, EED, and EZH2, is also important for promoting neurogenesis [84,85,86]. In vivo knockout of *Rnf2* (a component of the repressive complex PRC1 that mediates the ubiquitination of lysine 119 of histone H2A (H2AK119)) beginning at E13.5 or *Ezh2* (the lysine methyltransferase that trimethylates H3K27 in the repressive PRC2 complex) at E12.5 prolong the neurogenic phase, at the expense of astrogliogenesis [65,84]. *Rnf2* knockouts continue to have increased numbers of cortical neurons as late as P6.5 [84]. These studies indicate that a reduction in H3K27me3 promotes neurogenesis at the expense of astrogliogenesis during the neurogenic/gliogenic switch.

As opposed to H3K27me3, decreases in DNA methylation promote astrogliogenesis at the expense of neurogenesis. Genetic knockdown or pharmacological inhibition of DNMT1 or DNMT3A leads to global DNA hypomethylation in the developing central nervous system and precocious astrogliogenesis at the expense of neurogenesis [72,87,88]. Similarly, a *MeCP2* loss of function mutation increases neural cell astrocyte production compared with wild-type *MeCP2*-expressing cells [89].

Based on these data, it appears that prior to the time the neurogenic/gliogenic switch is normally engaged, low histone acetylation and H3K27 methylation and high DNA methylation prevent gliogenesis. Studies demonstrate that genetic or pharmacological approaches that increase overall histone acetylation and H3K27 methylation promote gliogenesis in the E13.5–15.5 brain, and knockdown of DNA methyltransferases or methyl DNA binding proteins from conception or early in development promotes gliogenesis. 

The switch from the neurogenic to the gliogenic phase normally takes place between E18.5 and E19.0 in the rodent neocortex [85]. The first step in this process occurs through the expression of NOTCH ligands, such as the Delta-like (DLL) proteins, by young neurons [90]. Neuronally expressed NOTCH ligands bind NOTCH receptors on astrocyte-fated cells, leading to the activation of NOTCH signaling [90]. NOTCH activation promotes the expression of the transcription factor Nuclear factor I A (NFIA), which participates in the DNA demethylation of astrogliogenesis promoting genes such as *Gfap*, S100 protein, beta polypeptide (*S100b*), and aquaporin genes [90]. DNA demethylation primes astrogliogenesis genes for the second step of this process, transcriptional activation by extracellularly released pro-astrogliogenic cytokines, such as leukemia inhibitory factor (LIF) [71]. Pro-astrogliogenic cytokines activate the Janus Kinase/Signal Transducer and Activator of Transcription (JAK-STAT) signaling pathway [71]. Activated NOTCH and basic helix-loop-helix (bHLH) proteins, such as Hairy and Enhancer of Split (HES), together with pro-astrogliogenic cytokines, promote STAT phosphorylation. Phosphorylated STAT (pSTAT) is able to bind the pro-astrogliogenesis gene promoters that are demethylated in the first step of this process leading to increased gene expression [71]. Bone Morphogenetic Proteins (BMPs) also promote astrogliogenesis in part by inducing histone acetylation of pro-glial promoters, facilitating their expression [91].

Following the neurogenic to gliogenic switch, DNA methylation and methylated DNA-histone complexes also play a role in astrocyte maturation, as suggested by the fact that either the knockdown of DNMT1 or the presence of a *MeCP2* mutation hastens astrocytes to attain a more fully mature state [72,89]. Precociously differentiated astrocytes from *MeCP2* mutant mice fail to support the normal dendritic arborization of neurons containing wild-type *MeCP2* [92,93,94]. 

**Figure 1:**
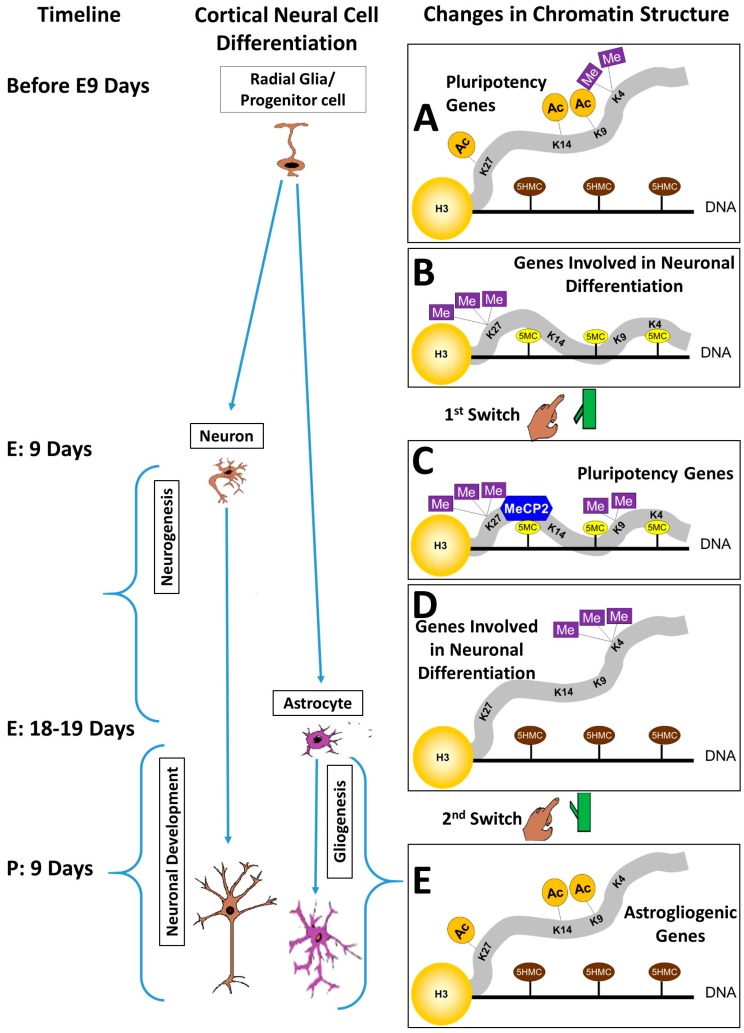
Cortical neural cell development and changes in chromatin structure. (**A**) Prior to embryonic day (E) nine neural precursor cells are in an undifferentiated state that is maintained by a combination of histone acetylation and histone lysine 4 (H3K4) methylation associated with increased gene expression of pluripotency genes, such as *Pou5f1* and *Nanog*; (**B**) repressive histone modifications, such as histone 3 lysine 27 methylation (H3K27me2/3) and DNA methylation (5-methylcytosine (5mC)) prevent expression of genes that would lead to neuronal differentiation such as high-mobility group A (HMGA) family of DNA-binding proteins and *Dlx* homeobox genes; (**C**) at approximately E9, a chromatin regulation switch is flipped between pluripotency to a neurogenic phase of brain development. Pluripotency gene promoters are switched off by restrictive histone modifications, DNA methylation, and the methyl-CpG binding domain protein MeCP2 binding to 5mC; (**D**) Neurogenic genes are turned on through the removal of methyl groups from H3K27, the addition of methyl groups to lysine 4 of H3 (H3K4me2/3), and the conversion of repressive 5mC to 5-hydroxymethylcytosine (5hmC); (**E**) at approximately E18–19 in the neocortex, the chromatin regulation switch is flipped once again, this time turning off neurogenic genes in neural cell precursors and turning on astrogliogenic genes. Histone acetylation and DNA demethylation turns on astrogliogenesis. By postnatal day (P) 7 most neurons have reached their final location in the neocortex, while gliogenesis continues. Orange: histone acetylation; purple: histone methylation; blue: proteins binding DNA; yellow: DNA methylation; brown: DNA 5-hydroxymethylation.

These studies indicate that the timing of the switch from neurogenesis to astrogliogenesis is tightly regulated by a series of epigenetic events. Prior to astrogliogenesis, high DNA methylation, MeCP2 binding to methylated DNA, and low H3K27 methylation, prevent the premature activation of glial cell gene expression. At E18–19 in the rodent neocortex, young neurons express NOTCH, which activates a cascade of events leading to DNA demethylation and increased histone acetylation of glial genes, facilitating their expression. After astrogliogenesis begins, DNA methylation and MeCP2 continue to play important roles in preventing precocious maturation of astrocytes. An environmental stimulus, such as alcohol, that could perturb these epigenetic events would therefore likely lead to abnormal differentiation or maturation of neurons and astrocytes (Figure 1).

## 5. Abnormal Differentiation in FASD

Alcohol can profoundly perturb the well-orchestrated events that determine the number, distribution, morphology, and functioning of neurons and astrocytes. Low doses of alcohol have been shown to promote stem cell expansion, while higher concentrations slow proliferation [95,96,97,98,99]. Several cell culture studies indicate that alcohol exposure inhibits neurogenesis [100], while hastening neuronal differentiation [100,101]. During the neurogenic period, alcohol causes a decrease in a variety of neuronal populations including hippocampal CA1 pyramidal cells [102,103,104,105,106,107,108,109,110,111], cerebellar Purkinje cells [112,113,114,115], and cerebral cortical neurons [116,117]. These effects may be caused by both a disruption of neuronal generation when exposure occurs earlier in gestation, and neuronal apoptosis when exposure occurs later in gestation during defined sensitive periods [103,116,118,119,120,121,122]. 

Alcohol also differentially affects specific neuronal populations. Several studies indicate that prenatal alcohol reduces the density and functioning of GABA neurons [123,124,125,126,127,128], and increases glutamatergic neurons [100]. These changes may be related to alcohol induction of pro-glutamatergic genes, such as Paired box 6 (*Pax6*), Neurogenin 2 (*Neurog2*), and Neurogenic Differentiation-1 (*Neurod1*) and inhibition of pro-GABAergic Mammalian Achaete Scute Homolog-1 (*Ascl1*) levels [100]. Additionally, prenatal alcohol exposure alters the distribution and morphology of neurons [101,129]. Ectopic distribution and density of neuronal synapses generated late in gestation have been reported [116,130,131,132,133,134]. 

In cell culture experiments, short-term (48 h) alcohol treatment of neural stem cells (NSC) decreases the size of astrocytes, but long-term treatment (eight days) increases their size [134]. In astrocytoma cell cultures, 24 h alcohol treatment decreases glial cell proliferation [135]. In vivo, prenatal alcohol exposure also decreases glial growth and proliferation and GFAP expression [136,137]. However, neonatal alcohol exposure increases GFAP expression [138,139,140,141]. 

Based on these reports, it is clear that ethanol alters both neuronal and astrocyte differentiation and maturation. Prior to the neurogenesis phase of neural cell development, ethanol causes premature neuronal differentiation, but exposure during the neurogenesis phase induces neuronal apoptosis. Additionally, ethanol alters the neuronal cell types produced, with increased numbers of glutamatergic neurons and fewer GABAergic neurons. Further, ethanol alters the morphology of neuronal processes. Finally, in utero ethanol exposure also affects astrocyte development. Ethanol reduces glial cell growth and proliferation. Overall, it appears that ethanol has profound effects on neural cell differentiation and maturation. However, the underlying molecular mechanisms alcohol disrupts remain to be fully established. 

## 6. FASD, Cell Fate, and Chromatin Structure

The coordinated chromatin remodeling events that lead to the neuroepithelial cell lineage and ultimately to neuron and astrocyte generation are acutely sensitive to alcohol. Over the last decade, alcohol-mediated effects on DNA methylation and histone modifications have been increasingly characterized in cell, animal, and clinical reports. Studies have begun investigating the effects of alcohol-induced histone modifications, specifically regarding their roles in differentiation. One challenge in determining the effects of alcohol on neural cell development is the variations that occur due to differences in dose and time of exposure in different studies. For example, Veazey et al. measured acute alcohol-induced changes in multiple histone modifications across 22 gene promoters in NSCs. They reported different effects on H3K4me3, H3K9ac, H3K9me2, and H3K27me3 levels depending on alcohol dose (160 mg/dL vs. 320 mg/dL), and whether alcohol was present at the time of culture harvest vs. four days following alcohol exposure [142]. The impact of alcohol on the expression and function of chromatin regulators may also shift considerably with age. Early gestational alcohol exposure produces different effects than alcohol exposure during the equivalent of the third human trimester (which, in rodents, corresponds to the first nine postnatal days). In addition, histone and DNA modifications are very dynamic during normal development [143]. These developmental changes may lead to alcohol exposure producing differences in adulthood that are not apparent at adolescence or vice versa. For example, prior to adulthood, Solute Carrier Family 17 Member 6 (*Slc17a6*) mRNA expression is highly expressed in both alcohol- and non-alcohol exposed mice. However, in adulthood, when *Slc17a6* mRNA expression normally declines, alcohol-exposed mice continue to express *Slc17a6* mRNA at high levels. The abnormally high *Slc17a6* mRNA expression is associated with lower promoter DNA methylation in alcohol-exposed mice [144]. 

An additional challenge to understanding the effects of alcohol on epigenetic parameters and neural cell development is the fact that different neural cell types are characterized by different histone modification and DNA methylation levels. As previously mentioned, alcohol alters neural cell development, leading to changes in proportions of neural cell populations. While this review proposes that alcohol alters epigenetic parameters contributing to differences in neural cell fractions, it is also possible that observed changes in epigenetic measures are a reflection of the different numbers of neural cells present in the brain following alcohol exposure. 

Differences in DNA methylation and histone acetylation have been reported in various neural cell types. Mature astrocytes have lower global levels of histone acetylation relative to neurons [76]. In addition, neurons have lower global DNA methylation levels compared with non-neuronal cells [145]. Even among neuronal subtypes, there are differences in DNA methylation characteristics. For example, DNA methylation levels of Vasoactive intestinal polypeptide-expressing (VIP) GABA interneurons are more similar to glia than excitatory or parvalbumin interneurons. This same correlation between glia and VIP neurons was reported in ATAC-seq experiments, in which nucleosome free regions are characterized [146]. It was also reported that CpH methylation is significantly more abundant in neuronal vs. non-neuronal cells [147], and CpH methylation is most abundant in parvalbumin positive GABA interneurons [146]. Therefore, until studies emerge in which chromatin changes are measured specifically by cell type, it is difficult to ascertain whether reported alcohol-induced changes in epigenetic parameters are correlative or causative in relation to differences in neural cell populations.

## 7. FASD and DNA Methylation

The lifelong central nervous system phenotypic and gene expression changes caused by prenatal alcohol exposure’s effects on chromatin structure are often difficult to discern. This is because there are numerous genes impacted by alcohol in the brain and behavioral phenotypes are often subtle. Examples of more obvious phenotypes directly connecting alcohol-induced DNA methylation and gene expression changes do exist [1]. For example, prenatal alcohol exposure increases Agouti viable yellow (*A^vy^*) intercisternal A-particle (IAP) DNA methylation, producing an increase in the number of offspring with a pseudoagouti coat color and reduced body weight [148]. Similarly, a single alcohol treatment on E9 reduced methylation of the maternally imprinted Insulin Like Growth Factor 2 (*Igf2*) gene in whole embryos resulting in higher prenatal mortality, lower prenatal growth, and digit and vertebral malformations [149]. The manner and degree to which DNA methylation affects gene expression and phenotype is often not as straightforward as it is for *A^vy^* or imprinted genes, especially in the central nervous system. However, from these studies it is apparent that there are instances in which prenatal alcohol’s effects on DNA methylation can produce lifelong phenotypic changes. 

Altered DNA methylation levels in FASD are likely, at least partially, a consequence of changes in one-carbon metabolism and its involvement with S-adenosylmethionine (SAM) production [150]. SAM serves as a co-substrate for DNA and histone methylation, among many other enzymatic processes [150]. SAM is synthesized, in part, from precursors including folic acid and choline, where homocysteine is a product and precursor of SAM metabolism [151]. Alcohol inhibits folic acid absorption and reduces the ability of methionine synthase to convert homocysteine to methionine, which is often subsequently converted to SAM [152,153,154,155]. The fact that maternal dietary folate and choline supplementation have been shown to attenuate the effects of prenatal alcohol exposure supports the notion that a reduced supply of methyl donors is associated with FASD [156,157]. 

In cell culture experiments, alcohol has profound effects on DNA methylation. Primary astrocyte cultures treated for 24 h with alcohol show reduced DNMT expression and activity [158]. Similarly, 48 h of alcohol treatment of embryonic fibroblasts decreased DNA methylation levels, DNMT expression, and DNMT activity [159]. By contrast, a 48-h treatment of NSC increased DNMT activity and expression [97], but did not affect 5mC or 5hmC levels [134]. Longer-term treatment (eight days) increased global levels of 5mC without affecting 5hmC levels, and alcohol withdrawal decreased 5hmC in these cells [134]. 

The impact of DNA methylation changes on gene expression is partly dependent upon methyl-CpG binding proteins, such as MeCP2. In embryonic fibroblasts and primary neuronal cultures, alcohol decreased MeCP2 protein expression [159,160]. These results contrasted with studies using NSCs in which short-term (48 h) or long-term (eight days) treatment with alcohol increased MeCP2 mRNA and protein expression, while alcohol withdrawal reduced MeCP2 levels [134]. In this study, none of the treatment conditions affected the ultimate numbers of neural cell types [134]. However, all three conditions increased neurite outgrowth [134]. 

In animal experiments, a growing body of literature suggests that prenatal alcohol exposure decreases DNA methylation when measured during early life (prenatally or neonatally), but increases methylation when measured during adolescence or adulthood, whether measured in the whole embryo, the hippocampus, or the neocortex [161,162,163]. Alcohol treatment also reduces prenatal and neonatal DNMT expression and activity [158,162]. Similarly, in mice in which alcohol exposure starts from E5 or E6 and continues to E15–16, there are lower hippocampal, striatal, and cortical MeCP2 levels at E17 and at five weeks [129,164]. In contrast, prenatal or neonatal alcohol exposure results in an increase in global DNA methylation, DNMT activity, and DNMT expression in the hippocampus, neocortex, and hypothalamus of adolescent or adult rodents [165,166,167,168,169,170]. Prenatal and postnatal alcohol exposure leads to increased hippocampal, neocortical, and hypothalamic MeCP2 levels at P8 and adulthood [168,170,171,172] (Table 1). 

Interestingly, there are reports that central nervous system genes are particularly sensitive to the effects of prenatal alcohol on DNA methylation. In genome-wide studies of children with prenatal alcohol exposure, DNA methylation changes are especially abundant at neurodevelopmental genes in buccal epithelial cells and blood [173,174]. Alcohol treatment preferentially alters DNA methylation levels at genes related to neuronal function in NSCs [175]. Similarly, prenatal alcohol treatment of mice alters DNA methylation in the brain at genes participating in neurological, behavioral, and psychological disorder networks [176]. 

Studies examining the methylation status at FASD candidate genes have also been undertaken. Increased DNA methylation and MeCP2 binding at the Pro-opiomelanocortin (*Pomc*) and the serotonin transporter (*Slc6a4*) genes in the hypothalamus [168,169,172,181] and the *Gfap* promoter in whole brain samples have been reported [178]. Decreased DNA methylation also occurs at the *Slc17a6* gene promoter in the hippocampus and the Plasminogen Activator, Tissue Type (*Plat*) gene in cultured astrocytes [144,158]. Notably, a change in DNA methylation does not always correspond to changes in gene expression in predictable ways. For example, during the third trimester equivalent in rodents, alcohol exposure is associated with DNA methylation and corresponding mRNA changes in only eight genes. In contrast to what might be anticipated, increased DNA methylation is associated with increased expression of five of these eight genes [166]. 

Taken together, it appears that the immediate effects of prenatal alcohol exposure are to decrease global DNA methylation and MeCP2 levels [129,159,160,161,162,164,189,190], while the longer lasting effects, observed during adolescence or adulthood, are an increase in DNA methylation and MeCP2 levels [165,166,168,171,172]. Based on the neurodevelopmental literature, a global reduction in DNA methylation and MeCP2 levels might be expected to promote astrocyte differentiation, and possibly delay or decrease neurogenesis [72,89]. In this way, alcohol would be similar to DNMT inhibitor treatment, which is known to induce differentiation along the astrocyte lineage [87]. This was reported in previous studies in which alcohol induces premature transformation of radial glial cells to astrocytes [139,191,192,193]. Further support is indicated by the fact that choline and folate supplementation of maternal or alcohol-exposed pups attenuates the behavioral effects of fetal alcohol exposure and promotes neural stem cell differentiation into neurons both in vitro and in vivo [87,157,194,195]]. Moreover, when administered during late gestation (after E18), alcohol induces DNA hypomethylation and lower MeCP2 levels. This may lead to advanced differentiation of astrocytes already committed to that cell fate [72,87,88]. These precociously mature astrocytes would likely not be able to support normal neuritogenesis in neighboring neurons [92,196]. In support of this hypothesis, the pretreatment of astrocyte cultures with alcohol reduces neurite outgrowth of subsequently co-cultured neurons [197,198].

## 8. FASD and Histone Modifications

Cell culture experiments reveal that the effects of ethanol on histone modifications are in part dependent upon the dose of ethanol, as well as whether ethanol is present at the time of culture harvesting. Prior ethanol exposure (prolonged withdrawal) in NSC cultures shifts chromatin to a more condensed state compared to when ethanol is still present [142]. Further, lower alcohol doses increase histone acetylation, while higher doses decrease it [142]. In accordance with higher doses and alcohol withdrawal from cell culture inducing more condensed chromatin, high-dose alcohol treatment of neural progenitor cells (NPCs) induces the expression of the H3K9 methytransferase *Ehmt1* and decreases expression of the H3K4 methyltransferase complex protein Absent, Small, or Homeotic-Like (*Ash2l*) transcripts, while withdrawal increases *Ehmt2*, *Setdb1*, *Eed*, and *Ezh2* [142,183]. 

Alcohol exposure during development also alters histone modifications in vivo. The expression of *Hdac1* mRNA decreases four hours following alcohol exposure at P7 [199], while *Hdac2* expression increases in adulthood in the hypothalamus [169]. Prenatal alcohol exposure reduces histone acetylation and *Crebbp* mRNA levels in the hypothalamus in adulthood, while neonatal exposure (from P2–12) reduces histone acetylation and CREBBP protein in the cerebellum within 1 h after the final exposure on P2–10 but no change relative to the control was seen at P12 [169,185]. 

While the effects of prenatal alcohol on transient histone acetylation marks vary by model and paradigm, prenatal alcohol primarily promotes increases in global levels of restrictive histone methyl marks. Prenatal (E7–21) or early postnatal (P7) alcohol exposures increase global levels of H3K9me2 relative to total H3 and H3K27me2 in the hypothalamus, hippocampus, and neocortex, while reducing H3K4me2/3 [168,169,187,200]. 

Consistent with these findings, fetal or early postnatal alcohol exposure decreases mRNA levels of H3K4 methyltransferase *Setd7* and increases mRNA levels of H3K9 methyltransferases *Ehmt2* and *Setdb1*, in the hypothalamus in adulthood, and in the hippocampus and neocortex at P8 [168,169,170,187]. 

The functional consequence of an alcohol-induced increase in repressive histone methyl marks is generally, as predicted, decreased gene expression. In a recent meta-analysis of microarray studies, the authors concluded that prenatal alcohol exposure generally leads to reduced levels of gene expression [201]. In a transcriptome-wide study that employed whole mouse brain samples of adult mice exposed during the equivalent of the first, second, and third trimesters of pregnancy, 69%, 77%, and 96%, respectively, of the genes that were differentially expressed were downregulated in the alcohol groups when compared with control groups [186]. On the other hand, a recent study that used DNA isolated by chromatin immunoprecipitation to probe a microarray demonstrated an overall decrease in H3K27me3 levels following third-trimester alcohol exposure at adulthood in the hippocampus (Table 2) [166]. They also reported that the expression of only 60 genes in the adult hippocampus differed from controls following alcohol exposure during the third trimester equivalent. The mRNA levels of two-thirds of these genes were increased [166]. The same study reported that 45 of the alcohol-affected genes exhibited no changes in promoter H3K4me3 or H3K27me3 levels; 11 genes displayed changes in H3K4me3 and four in H3K27me3. Of the 11 genes displaying changes in H3K4me3, five followed the pattern of expression generally associated with this histone modification (i.e., increased H3K4me3 associated with increased expression, or decreased H3K4me3 associated with decreased expression), while the other six did not. All four genes with changes in H3K27me3 displayed decreased H3K27me3 and increased gene expression [166]. This study supports the notion that one or even a few marks in the histone tails do not always determine whether a gene is expressed or not. Gene expression is the result of a more complex interaction between multiple histone tail modifications and cross-talk with intracellular signaling systems [202,203].

Overall, it appears that prenatal alcohol exposure increases histone PTMs associated with condensed chromatin. High doses of ethanol increase H3K9 and H3K27 methyltransferase expression and H3K9 and H3K27 methylation in cell culture and in animal models. Conversely, ethanol reduces H3K4 methyltransferases and H3K4 methylation levels. As a consequence, overall transcriptional activity is generally reduced in FASD models. We suggest that the functional impact of these changes is to alter neural cell fate. As the genes affected by prenatal alcohol chromatin changes are identified, it may become possible to identify children with FASD earlier in development, and to develop pharmacotherapies to address the associated deficits.

## 9. Conclusions

From the review of the FASD literature, it is apparent that prenatal alcohol exposure alters neural cell differentiation as well as chromatin structure. Future studies associating epigenetic changes induced in well-characterized alcohol models during brain development with alterations in cell genesis and differentiation would constitute an interesting new research direction that may lead to a better understanding of the origins of FASD pathophysiology. These types of studies are particularly important considering that even small variations in the developmental stage at the time of administration, doses of alcohol, or duration of alcohol exposure can lead to different epigenetic responses. This may ultimately lead to a better understanding of the mechanisms behind the cognitive and behavioral manifestations of FASD. In addition, studying these changes in neurons and glial cells separately may provide additional information regarding the impact of alcohol on different brain cells.

There is an emerging literature that suggests that alcohol exposure may induce histone modifications associated with condensed chromatin both globally and specifically at genes involved in neurogenesis in models of second-trimester alcohol exposure, a time when neurogenesis peaks in mammals [142,183]. Restrictive histone marks, such as H3K27me3, suppress neurogenesis to maintain pluripotency early in gestation [65,84,85,86]. Therefore, an early alcohol exposure-induced increase in H3K27 and decrease in H3K4 methylation might suppress neurogenesis. This anti-neurogenic state is further exacerbated by an alcohol-induced reduction in DNA methylation and in the methyl DNA binding protein MeCP2 expression [129,159,161,162,164]. Reduced levels of DNA methylation and MeCP2 would be expected to promote astrogliogenesis at the cost of neurogenesis and to hasten the maturation of astrocytes. In addition to impairing neuronal production, precociously mature astrocytes might not be able to promote normal neuritogenesis. Altered synaptic morphology resulting from altered neuritogenesis could impair neuronal communication manifesting as long-term potentiation and memory deficits. 

An obvious challenge to the development of pharmacological treatments for FASD is that, while the insult occurs prenatally, any potential treatment would likely be administered later, after most of the damage has already occurred. Future studies aimed at identifying chromatin abnormalities in peripheral tissues could lead to earlier diagnosis and treatment. Laufer et al. found that DNA methylation of certain CNS genes, such as procadherin, are altered in the buccal swabs of children born with FASD, possibly indicating that some FASD-related DNA methylation perturbations are present in both the CNS and periphery [174]. In addition, by determining which neurodevelopmental processes are perturbed but remain amenable to pharmacological intervention postnatally, it may be possible to develop medications that mitigate the damage caused by prenatal alcohol exposure. In rodents, choline administered during the equivalent of the third trimester of pregnancy in humans or later mitigates some of the cognitive phenotypes associated with FASD, and prevents DNA hypermethylation in the hippocampus and cortex. On the other hand, results from human clinical trials have been somewhat mixed, with some studies showing benefits for affected children and others not finding any beneficial effects [165,168,204,205,206]. Another possible therapeutic approach would be to target cell populations that are still differentiating in humans after birth. Astrocyte differentiation continues during the early postnatal period in humans, and, as examined in this review, is highly reliant upon histone modifications and DNA methylation. Therefore, medications targeting astrocyte development may be especially promising. As the effects of prenatal alcohol on chromatin structure are elucidated, it may become possible to develop drugs that alter histone modifications and/or DNA methylation to restore normal postnatal differentiation and maturation of neural cells, thereby minimizing the neurobehavioral impact of alcohol exposure. 

## Figures and Tables

**Table 1:** Fetal alcohol induced changes in DNA methylation.

**5-methylcytosine (5mC)**							
**Global or Gene Specific**	**Increase or Decrease**	**Time Alcohol Administered**	**Time Studied**	**Brain Region/Cell Culture**	**Species**	**Sex**	**Reference**
**Global**	↓		48 h	embryonic fibroblasts	mice	M	[159]
	↓	E8.25	E10	Neural tube	mice	U	[175]
	↓	E9–11	E11	whole embryo	mice	B	[161]
	NC	E5–16	E17	ammonic neuroepithelium	mice	B	[164]
	↑	E5–16	E17	intermediate zone	mice	B	[164]
	↑	E5–16	E17	hippocampus CA1	mice	B	[164]
	↓	P7	P8	hippocampus	mice	B	[162]
	↓	P7	P8	neocortex	mice	B	[162]
	↑	P2–10	P21	Prefrontal cortex	rats	B	[165]
	↑	P2–10	P21	hippocampus	rats	B	[165]
	NC	E7–21	P60–80	hypothalamus	rats	B	[169]
**Genome-wide**	mostly ↓		48 h	NSC		B	[163]
	↑↓	E8–10 *	E10	whole embryo	mice	B	[177]
	mostly ↑	P4 & P7	P70	Hippocampus	mice	M	[166]
	↑↓		5–18 yo	Buccal Epithelial Cells	human	B	[173]
**Cell cycle genes**	↑		48 h	NSC		F	[97]
**MeCP2 promoter**	↑	D0–2	D2	NSC	mice	B	[134]
	↓	D0–8	D8	NSC	mice	B	[134]
**Igf2**	↓	E9	E9	whole embryo	mice	B	[149]
**Gfap promoter**	↑	E1–21	E21	whole brain	rats	B	[178]
**Plat promoter**	↓		24 h	primary cortical astrocytes	rats	B	[158]
**Imprinted genes (H19 and IG-DMR)**	↓	correlated with alcohol drinking		sperm	human	M	[179]
**KCNQ1OT1**	↓		1–26 yo	blood	human	B	[180]
**PEG3 promoter**	↓		1–26 yo	blood	human	B	[180]
**Slc6a4 promoter**	↑	E1–21	P55	hypothalamus	rats	F	[181]
**Pomc promoter**	↑	E7–21	P60–80	hypothalamus	rats	B	[169]
**Gm9268 promoter**	↑	E0.5–8.5	P28	hippocampus	mice	M	[182]
**Vpreb2 promoter**	↑	E0.5–8.5	P28	hippocampus	mice	M	[182]
**Olfr601 promoter**	↓	E0.5–8.5	P28	hippocampus	mice	M	[182]
**Slc17a6 promoter**	↓	E0.5–8.5	P120	hippocampus	mice	M	[144]
**5-hydroxymethylcytosine (5hmC)**							
**Global or Gene Specific**	**Increase or Decrease**	**Time Alcohol Administered**	**Time Studied**	**Brain Region/Cell Culture**	**Species**	**Sex**	**Reference**
**Global**	↓	E5–16	E17	ammonic neuroepithelium	mice	B	[164]
	↓	E5–16	E17	intermediate zone	mice	B	[164]
**DNMT Expression/Activity**							
**DNMT Isoform mRNA, Protein, Activity**	**Increase or Decrease**	**Time Alcohol Administered**	**Time Studied**	**Brain Region/Cell Culture**	**Species**	**Sex**	**Reference**
**Dnmt1 mRNA**	↓		48 h	embryonic fibroblasts	mice	M	[159]
	↑		5D	neurospheres	mice	B	[183]
	↑	D1–3	D7	neurospheres	mice	B	[142]
	NC		24 h	primary cortical astrocytes	rats	B	[158]
	↓	P90–155	P155	sperm	rats	M	[184]
	↓	P7	P8	hippocampus	mice	B	[162]
	↑	P7	P8	neocortex	mice	B	[162]
	↑	E7–21	P60–65	hypothalamus	rats	M	[168]
**DNMT1 protein**	↓		48 h	embryonic fibroblasts	mice	M	[159]
	↑		48 h	NSC		F	[97]
	NC		24 h	primary cortical astrocytes	rats	B	[158]
	NC	E6–15	P35	striatum	mice	U	[129]
	↓	P7	P8	hippocampus	mice	B	[162]
	NC	E6–15	P35	cortex	mice	U	[129]
	↓	P7	P8	neocortex	mice	B	[162]
	↑	E7–21	P60–65	hypothalamus	rats	M	[168]
**Dnmt3a mRNA**	↑		48 h	embryonic fibroblasts	mice	M	[159]
	NC		24 h	primary cortical astrocytes	rats	B	[158]
	↓	P7 (high dose)	P8	hippocampus	mice	B	[162]
	↑	P7 (low dose)	P8	hippocampus	mice	B	[170]
	↓	P7 (high dose)	P8	neocortex	mice	B	[162]
	↑	P7 (low dose)	P8	neocortex	mice	B	[170]
**DNMT3A protein**	↓		48 h	embryonic fibroblasts	mice	M	[159]
	↓		24 h	primary cortical astrocytes	rats	B	[158]
	↓	P7 (high dose)	P8	hippocampus	mice	B	[162]
	↑	P7 (low dose)	P8	hippocampus	mice	B	[170]
	↓	P7 (high dose)	P8	neocortex	mice	B	[162]
	↑	P7 (low dose)	P8	neocortex	mice	B	[170]
	↑	E7–21	P60–65	hypothalamus	rats	M	[168]
**Dnmt3b mRNA**	↑		48 h	embryonic fibroblasts	mice	M	[159]
**DNMT3B protein**	↓		48 h	embryonic fibroblasts	mice	M	[159]
**DNMT activity**	↑		48 h	NSC		F	[97]
	↓		24 or 48 h	primary cortical astrocytes	rats	B	[158]
	↓	E9–11	E11	whole embryo	mice	B	[161]
	↑	E1-P10	P21	hippocampus	rats	B	[167]
**MBD Expression**							
**MBD Isoform mRNA/Protein**	**Increase or Decrease**	**Time Alcohol Administered**	**Time Studied**	**Brain Region/Cell Culture**	**Species**	**Sex**	**Reference**
**MeCP2 mRNA**	↑		48 h	embryonic fibroblasts	mice	M	[159]
	↑	D0–2	D2	NSC	mice	B	[134]
	↑	D0–8	D8	NSC	mice	B	[134]
	↓	D0–2	D8	NSC	mice	B	[134]
	↓	D3–13	D13	primary cortical neurons	mice	B	[160]
	↓	D3–8	D13	primary cortical neurons	mice	B	[160]
	↑	E7–21	P60–65	hypothalamus	rats	M	[168]
	NC	P7 (low dose)	P8	hippocampus	mice	B	[170]
	NC	P7 (low dose)	P8	neocortex	mice	B	[170]
**MeCP2 protein**	↓		48 h	embryonic fibroblasts	mice	M	[159]
	↑	D0–2	D2	NSC	mice	B	[134]
	↑	D0–8	D8	NSC	mice	B	[134]
	↓	D0–2	D8	NSC	mice	B	[134]
	↓	D3–13	D13	primary cortical neurons	mice	B	[160]
	↓	D3–8	D13	primary cortical neurons	mice	B	[160]
	↓	E6–15	P35	striatum	mice	U	[129]
	↓	E5–16	E17	hippocampus	mice	B	[164]
	↑	P7 (low dose)	P8	hippocampus	mice	B	[170]
	↑	E8–21	Adult	hippocampus	rats	M	[171]
	↓	E6–15	P35	cortex	mice	U	[129]
	↑	P7 (low dose)	P8	neocortex	mice	B	[170]
	↑	E7–21	P60–65	hypothalamus	rats	M	[168]
**Mbd2 mRNA**	↓		48 h	embryonic fibroblasts	mice	M	[159]
**MBD2 protein**	↓		48 h	embryonic fibroblasts	mice	M	[159]
**Mbd3 mRNA**	↓		48 h	embryonic fibroblasts	mice	M	[159]
**MBD3 protein**	↓		48 h	embryonic fibroblasts	mice	M	[159]

Abbreviations: 5mC, 5-methylcytosine; 5hmC, 5-hydroxymethylcytosine; B, Both sexes; CA1, Cornu Ammonis-1; D, Number of days cultured; DNMT1, DNA methyltransferase-1; DNMT3A, DNA methyltransferase-3A; DNMT3B, DNA methyltransferase-3B; E, embryonic day; F, Female; Gfap, Glial fibrillary acidic protein; h, hours; IG-DMR; intergenic-differentially methylated region; Igf2, Insulin Like Growth Factor 2; KCNQ1OT1, KCNQ1 opposite strand/antisense transcript 1; M, Male; MBD, Methyl-CpG binding domain; MeCP2, methyl CpG binding protein 2; NC, No change; NSC, neural stem cells; Olfr601, olfactory receptor 601; P, postnatal day; PEG3, Paternally Expressed 3; Plat, Plasminogen Activator, Tissue Type; Pomc, Pro-opiomelanocortin; Slc17a6, Solute Carrier Family 17 Member 6; Slc6a4, serotonin transporter; U, Unclear; Vpreb2, pre-B lymphocyte gene 2; yo, years old; * Embryo treated ex vivo.

**Table 2:** Fetal alcohol induced changes in histone modifications.

**Histone Acetylation**							
**Global or Gene Specific**	**Increase or Decrease**	**Time Alcohol Administered**	**Time Studied**	**Brain Region/Cell Culture**	**Species**	**Sex**	**Reference**
**Global**	↓	E7–21	P60–80	hypothalamus	rats	M	[168]
	↓	P2–10	P2–10	cerebellum	rats	B	[185]
	NC	P2–12	P12	cerebellum	rats	B	[185]
**22 Growth factor genes**	↑	D1–3 (low dose)	D3	NSC	mice	B	[142]
	↓	D1–3 (high dose)	D3	NSC	mice	B	[142]
	↑	D1–3 (low dose)	D7	NSC	mice	B	[142]
	↓	D1–3 (high dose)	D7	NSC	mice	B	[142]
**Ehmt2 promoter**	↑	P7 (low dose)	P8	neocortex	mice	B	[170]
**Cnr1 promoter**	↑	P7	P8	neocortex, hippocamus	mice	B	[177]
**Dlx2 Promoter**	↑	E7	E17	neocortex	mice	B	[142]
**HAT/HDAC Expression**							
**HAT/HDAC isoform mRNA/protein**	**Increase or Decrease**	**Time Alcohol Administered**	**Time Studied**	**Brain Region/Cell Culture**	**Species**	**Sex**	**Reference**
**Crebbp mRNA**	↓	E7–21	P60–80	hypothalamus	rats	B	[169]
**CREBBP protein**	↓	P2–10	P2–10	cerebellum	rats	B	[185]
**CREBBP protein**	NC	P2–12	P12	cerebellum	rats	B	[185]
**Hdac1 mRNA**	↓	P7	P7	whole brain	mice	M	[186]
**Hdac2 mRNA**	↑	E7–21	P60–80	hypothalamus	rats	B	[169]
**H3K4 Methylation**							
**Global or Gene Specific**	**Increase or Decrease**	**Time Alcohol Administered**	**Time Studied**	**Brain Region/Cell Culture**	**Species**	**Sex**	**Reference**
**Global**	↓	E7–21	P60–65	hypothalamus	rats	M	[168]
**22 Growth factor genes**	↑	D1–3 (low dose)	D3	NSC	mice	B	[142]
	↓	D1–3 (high dose)	D3	NSC	mice	B	[142]
	↑↓	D1–3 (low dose)	D7	NSC	mice	B	[142]
	↑↓	D1–3 (high dose)	D7	NSC	mice	B	[142]
**Genome-wide**	↑↓	P4 & P7	P70	Hippocampus	mice	M	[166]
**Slc17a6 promoter**	↑	E0.5–8.5	P120	hippocampus	mice	M	[144]
**Sox2 Promoter**	↓	D1–5	D5	neurospheres	mice	B	[183]
**Dlx2 Promoter**	↓	D1–5	D5	neurospheres	mice	B	[183]
**Pax6 Promoter**	↓	D1–5	D5	neurospheres	mice	B	[183]
**H3K4 Methyltransferase/Demethylase Expression**							
**HMT/Demethylase isoform mRNA/protein**	**Increase or Decrease**	**Time Alcohol Administered**	**Time Studied**	**Brain Region/Cell Culture**	**Species**	**Sex**	**Reference**
**Ash2l1 mRNA**	↓	D1–5	D5	neurospheres	mice	B	[183]
**Setd7 mRNA**	↓	E7–21	P60–80	hypothalamus	rats	M	[168]
**Kdm1b mRNA**	↓	D1–5	D5	neurospheres	mice	B	[183]
**H3K9 Methylation**							
**Global or Gene Specific**	**Increase or Decrease**	**Time Alcohol Administered**	**Time Studied**	**Brain Region/Cell Culture**	**Species**	**Sex**	**Reference**
**Global**	↑	E7–21	P60–65	hypothalamus	rats	M	[168]
**22 Growth factor genes**	↑	P7 (high dose)	P8	hippocampus	mice	B	[187]
	↑	P7 (high dose)	P8	neocortex	mice	B	[187]
	↓	D1–3 (low dose)	D3	NSC	mice	B	[142]
	↓	D1–3 (high dose)	D3	NSC	mice	B	[142]
	↑	D1–3 (low dose)	D7	NSC	mice	B	[142]
	↑	D1–3 (high dose)	D7	NSC	mice	B	[142]
**Cnr1 promoter**	↓	P7	P8	neocortex, hippocampus	mice	B	[188]
**Dlx2 Promoter**	↑	E7	E17	neocortex	mice	B	[142]
**Dlx3 Promoter**	↑	E7	E17	neocortex	mice	B	[142]

Abbreviations: Ascl1, Achaete-Scute Family BHLH Transcription Factor 1; Ash2l, Absent, Small, Or Homeotic-Like; B, Both sexes; Cnr1, Cannabinoid 1 receptor; CREBBP, CREB binding protein; D, Number of days cultured; Dlx1, Distal-Less Homeobox 1; Dlx2, Distal-Less Homeobox 2; Dlx3, Distal-Less Homeobox 3; E, embryonic day; EED, Embryonic Ectoderm Development; EHMT1, Euchromatic Histone-Lysine N-Methyltransferase 1; EHMT2, Euchromatic Histone-Lysine N-Methyltransferase 2; EZH2, Enhancer of Zeste 2; F, Female; H3K4, H3 lysine 4; H3K9, H3 lysine 9; H3K27, H3 lysine 27; HATs, Histone acetyltransferases; HDACs, Histone deacetylases; HMT, histone methyltransferase; Kdm1b, Lysine Demethylase 1b; M, Male; NC, No change; NSC, neural stem cells; P, postnatal day; Pax6, Paired Box 6; PRC2, Polycomb Repressive Complex-2; Slc17a6, Solute Carrier Family 17 Member 6; Setd7, SET domain containing (lysine methyltransferase) 7; SETDB1, SET Domain Bifurcated 1; Sox2, (sex determining region Y)-box 2.
